# Twenty-Four-Hour Mean Arterial Pressure and Pulse Pressure Are Associated with Hospitalization Duration at Delivery in Pregnant Women Referred for Cardiovascular Risk Assessment

**DOI:** 10.3390/jcm15135188

**Published:** 2026-07-02

**Authors:** Isabel Fernandez-Castro, Nestor Vazquez-Agra, Ana Alban-Salgado, Mariña Sanchez-Andrade, Susana Lopez-Casal, Anton Cruces-Sande, Emma Lopez-Prado, Oscar Seoane-Casqueiro, Antonio Pose-Reino, Alvaro Hermida-Ameijeiras

**Affiliations:** 1 Department of Internal Medicine, University Hospital Complex of Vigo, 36312 Vigo, Pontevedra, Spain; isabelfdzcastro@gmail.com; 2 Department of Internal Medicine, University Clinical Hospital of Santiago de Compostela, 15706 Santiago de Compostela, A Coruña, Spain; oscar.seoane.casqueiro@sergas.es (O.S.-C.); antonio.pose.reino@sergas.es (A.P.-R.); alvaro.hermida.ameijeiras@sergas.es (A.H.-A.); 3 Health Research Institute of Santiago de Compostela (IDIS), 15706 Santiago de Compostela, A Coruña, Spain; emmalopezprado@gmail.com; 4 Department of Psychiatry, Radiology, Public Health, Nursing and Medicine, Faculty of Medicine, University of Santiago de Compostela (USC), 15706 Santiago de Compostela, A Coruña, Spain; 5 Laboratory of Biochemistry and Clinical Analysis, University Hospital of Santiago de Compostela, 15706 Santiago de Compostela, A Coruña, Spain; ana.mercedes.alban.salgado@sergas.es; 6 Obstetric Service, University Hospital of Santiago de Compostela, 15706 Santiago de Compostela, A Coruña, Spain; marina.sanchez-andrade.santiso@sergas.es (M.S.-A.); susana.lopez.casal@sergas.es (S.L.-C.)

**Keywords:** hypertensive disorders of pregnancy, ambulatory blood pressure monitoring, mean arterial pressure, pulse pressure, length of hospital stay

## Abstract

**Background:** We evaluated the relationship between ambulatory blood pressure monitoring (ABPM) indices and prolonged hospitalization at delivery in pregnant women referred for ABPM assessment. **Methods:** This was a prospective observational study including 132 pregnant women, followed until delivery. Office and 24 h ABPM measurements were obtained early in pregnancy and mean arterial pressure (MAP) and pulse pressure (PP) were derived. Prolonged hospitalization was operationally defined as above-median length of stay in the study cohort (>4 days). Associations with prolonged hospitalization were evaluated using binary logistic regression models, while time to hospital discharge was analyzed using Cox proportional hazards regression. Discriminative performance was assessed using receiver operating characteristic (ROC) curve analysis, and time-to-event data were explored using Kaplan–Meier curves. **Results:** Prolonged hospitalization occurred in 33.3% of patients. In multivariate analyses, 24-hMAP (OR 1.64, 95% CI 1.05–2.55; *p* = 0.029) and 24-hPP (OR 1.71, 95% CI 1.06–2.74; *p* = 0.027) were independently associated with prolonged hospitalization. ROC curve analysis demonstrated AUC values of 0.866 and 0.859 for the 24-hMAP and 24-hPP models, respectively. At a 30% false-positive rate, the 24-hMAP model achieved approximately 90% sensitivity, whereas the 24-hPP model achieved approximately 90% specificity at a 30% false-negative rate. In time-to-event analyses, only 24 h MAP remained independently associated with time to hospital discharge (HR 0.75, 95% CI 0.62–0.89; *p* = 0.001). **Conclusions:** Both 24-hMAP and 24-hPP were independently associated with prolonged hospitalization whereas office BP-derived indices were not. These findings support that integrated ABPM-derived measures may provide complementary prognostic information beyond conventional BP assessments.

## 1. Introduction

Hypertensive disorders during pregnancy represent a major contributor to maternal and fetal morbidity, encompassing a spectrum from uncomplicated gestational hypertension to preeclampsia [1,2]. These conditions are characterized by complex hemodynamic and endothelial alterations that disrupt the physiological cardiovascular adaptations of pregnancy. In normal gestation, systemic vascular resistance decreases and blood pressure (BP) levels tend to decline, particularly during early and mid-pregnancy. However, this adaptive response may be attenuated in women with increased cardiovascular risk (CVR), leading to persistently elevated BP levels and a higher risk of adverse outcomes [3].

Ambulatory blood pressure monitoring (ABPM) has demonstrated superiority over office-based measurements in predicting pregnancy-related complications, particularly in hypertensive populations. By capturing circadian patterns and overall BP burden, ABPM provides a more comprehensive assessment of maternal hemodynamics. Despite this, the clinical implications of specific ABPM-derived indices—such as mean arterial pressure (MAP) and pulse pressure (PP), reflecting overall pressure load and arterial stiffness, respectively—remain incompletely characterized in this setting [4,5,6].

While previous studies have focused on the prediction of hypertensive complications, less attention has been paid to clinically relevant healthcare outcomes such as postpartum length of hospital stay [7,8,9]. Duration of hospitalization is a key indicator of disease severity, healthcare resource utilization, and maternal recovery [7,10]. Thus, identifying early predictors of postpartum length of stay may improve risk stratification and optimize clinical management.

In this context, we hypothesized that some specific ABPM-derived indices may provide additional prognostic information for length of hospital stay beyond conventional clinical variables. Therefore, we assessed the association between BP indices—both office-based and ambulatory—and length of hospital stay in pregnant women, as well as their diagnostic–predictive performance for prolonged hospitalization.

## 2. Materials and Methods

### 2.1. Study Design, Setting, and Participants

This was a prospective observational study conducted in the Department of Internal Medicine at the University Hospital of Santiago de Compostela during 2024. Pregnant women were consecutively enrolled according to the following inclusion criteria: (1) age equal to or higher than 18 years; (2) first trimester of pregnancy (<13 + 6 weeks of gestation); (3) absence of previous arterial hypertension (AHT) or presence of previously diagnosed, adequately controlled chronic hypertension; (4) referral from the obstetrics department for comprehensive CVR assessment, including 24 h ABPM, according to institutional protocols.

In the institutional clinical pathway, pregnant women were referred from Obstetrics to the Hypertension and Cardiovascular Risk Unit of Internal Medicine when clinical features suggested increased cardiovascular risk. Referral for ABPM was mainly driven by older maternal age, higher body mass index, higher baseline BP levels, previous or adequately controlled chronic hypertension, and/or previous preeclampsia. Therefore, ABPM was requested as part of a targeted cardiovascular assessment rather than as a population-based screening procedure, and the study cohort represents a clinically selected higher-risk subgroup of pregnant women. This pathway is summarized in Supplementary Figure S1 [11].

Exclusion criteria included previous smoking (within 6 months prior to pregnancy), any alcohol consumption, established cardiovascular disease, and contraindications to ABPM (including psychological–behavioral, sleep-related, or anatomical–physiological conditions) [11,12,13]. All participants were prospectively followed throughout pregnancy until hospital discharge after admission for delivery.

### 2.2. Clinical and Laboratory Variables

Clinical variables included maternal age, body mass index (BMI), and previous smoking status, which was recorded as a binary variable (yes/no). BMI was expressed in kg/m^2^ and derived from recorded weight and height [14]. Office BP measurements were obtained on the same day as ABPM, during the morning assessment and before initiation of the monitoring procedure. Measurements were performed in the seated position after at least 5 min of rest using a validated oscillometric device (WatchBP Office, Microlife Corporation, Widnau, Switzerland), according to STRIDE BP recommendations. Three consecutive measurements were obtained, the first measurement was discarded, and the average of the second and third measurements was used for analysis [15].

Arterial hypertension was defined according to the European Society of Hypertension (ESH) current clinical guidelines as office systolic BP (SBP) ≥ 140 mmHg and/or diastolic BP (DBP) ≥ 90 mmHg, or the use of antihypertensive medication. Chronic hypertension, also referred to as pre-existing hypertension, was defined as AHT diagnosed before pregnancy or before 20 weeks of gestation, or the use of antihypertensive medication for previously diagnosed hypertension before pregnancy. Gestational hypertension and preeclampsia were considered according to established obstetric criteria as newly developed hypertensive disorders occurring after 20 weeks of gestation [11,16]. Information on antihypertensive therapy at the time of the initial cardiovascular assessment and ABPM was obtained from clinical records and coded as a binary variable (yes/no), according to whether the patient was receiving any antihypertensive medication at that time. Diabetes mellitus (DM) was defined as a previous diagnosis according to the American Diabetes Association (ADA) guidelines [17].

On the same morning as office BP assessment and ABPM placement, venous blood was drawn at 08:00 AM after a 12 h fast. Uric acid was measured by colorimetric assay with the Atellica Solution platform (Siemens Healthcare Diagnostics, Tarrytown, NY, USA). Placental growth factor (PlGF) and soluble fms-like tyrosine kinase-1 (sFlt-1) were assessed by electrochemiluminescence immunoassay on a Cobas e411 analyzer (Roche Diagnostics, Zug, Switzerland), and the sFlt-1/PlGF ratio was subsequently derived.

### 2.3. Parameters of 24 h ABPM Collection

Twenty-four-hour ABPM was performed according to international recommendations for ambulatory blood pressure monitoring [18,19]. Validated oscillometric devices were used in accordance with STRIDE BP standards: Space-Labs 90207 (Space-Labs Inc., Redmond, WA, USA), Microlife WatchBP O3 (Microlife Corporation, Widnau, Switzerland), and Cardioline Walk 200b (AB Medica Group, S.A., Barcelona, Spain) [15].

ABPM was performed during the initial first-trimester cardiovascular risk assessment, around the standard first-trimester screening window (11 + 0 to 13 + 6 weeks of gestation). Only the first valid ABPM recording obtained during early pregnancy was used for the present analysis. Repeated ABPM assessments, when clinically performed later during pregnancy, were not included.

Measurements were scheduled at 20 min intervals during daytime and at 30 min intervals during nighttime. Daytime and nighttime periods were assigned according to each participant’s reported sleep and wake times. Recordings were accepted as valid when more than 70% of the expected readings were successfully obtained. During monitoring, participants completed a diary documenting sleep period, medication intake, and any symptoms or technical problems. If the participant reported clearly disrupted sleep during the recording, ABPM was repeated [15,20].

For each valid recording, mean systolic and diastolic BP values were calculated separately for the 24 h, daytime, and nighttime periods and reported as 24-hSBP, dSBP, nSBP, 24-hDBP, dDBP, and nDBP. Nocturnal systolic and diastolic dipping were expressed as the percentage decrease from daytime to nighttime BP [21].

### 2.4. Main Outcomes and Predictors

Two main outcomes were considered as follows: (1) prolonged hospitalization was operationally defined as above-median hospitalization duration, corresponding to a length of stay > 4 days in this cohort. This threshold was selected for analytical purposes and should not be interpreted as a universal clinical definition of complicated delivery or abnormal hospitalization; and (2) a time-to-event outcome was defined as the time from hospital admission to discharge after delivery, expressed in days. The event of interest was hospital discharge.

Given the large number of ABPM-derived indices and their high collinearity, a predefined analytical strategy was applied. Composite hemodynamic parameters were selected for multivariable analyses to reduce redundancy and improve model stability [19,22]. Therefore, MAP was chosen as an integrated measure of overall BP load, whereas PP was considered a surrogate marker of arterial stiffness [23,24]. For ABPM-derived variables, 24 h indices were prioritized over separate daytime and nighttime measurements, as they provide a comprehensive estimate of overall BP burden. Continuous BP variables were categorized into quartiles for regression analyses to account for potential non-linear relationships.

### 2.5. Ethical Statement

This study was conducted in accordance with the principles of the Declaration of Helsinki and Good Clinical Practice (GCP). All participants provided written informed consent prior to inclusion. The study protocol was approved by the Research Ethics Committee of Santiago-Lugo (code 2022/144).

### 2.6. Sample Size Calculation and Statistical Analysis

Statistical analyses were performed using SPSS version 22 (IBM Corp., Armonk, NY, USA). Categorical variables were expressed as absolute frequencies and percentages, whereas continuous variables were summarized as median and interquartile range (IQR). For the univariate analysis, qualitative and quantitative variables were compared using the chi-squared test and the Mann–Whitney U test, respectively.

To contextualize the selected nature of the study cohort, baseline characteristics of women who underwent ABPM were descriptively compared with those of pregnant women from the same institutional setting who did not undergo ABPM and had available baseline data. This comparison was performed only to describe the referral profile and external validity of the ABPM cohort; women who did not undergo ABPM were not included as a control group in the outcome analyses.

Continuous ABPM-derived variables were categorized into quartiles for regression analyses. This approach was selected to facilitate interpretation, reduce the influence of extreme values, and avoid imposing a strictly linear association between hemodynamic indices and hospitalization duration. In addition, this strategy was consistent with the structure of the multivariable models, in which most clinical covariates were categorical variables.

Associations with prolonged hospitalization were evaluated using binary logistic regression models, and results were expressed as odds ratios (ORs) with 95% confidence intervals (95% CIs). Prior to model building, potential confounding and interaction effects between variables of interest and relevant covariates were assessed. Variables with a *p*-value < 0.05 in univariate analyses were entered into the multivariable models, and a backward stepwise selection procedure (Wald method) was applied, with variables retained at *p* < 0.10. Model complexity was restricted according to the conventional rule of approximately 10 outcome events per variable to reduce the risk of overfitting [25]. Internal validation of the logistic regression models was additionally performed using bootstrap resampling with 1000 iterations to assess coefficient stability and confidence interval robustness for the main ABPM-derived BP indices. Model-derived predicted probabilities, as well as individual office and ambulatory blood pressure-derived indices, were used to construct receiver operating characteristic (ROC) curves, from which area under the curve (AUC), sensitivity, and specificity were obtained at clinically relevant thresholds.

Kaplan–Meier curves were estimated using the Kaplan–Meier method and compared using the log-rank test. Time to hospital discharge was analyzed using Cox proportional hazards regression models, and results were expressed as hazard ratios (HRs) with 95% confidence intervals (95% CIs). The proportional hazards assumption was assessed using time-dependent covariates and graphical methods. A two-sided *p*-value < 0.05 was considered statistically significant.

Sample size estimation was performed a priori for the primary time-to-discharge analysis using Schoenfeld’s method for Cox regression [26]. Assuming a two-sided alpha of 0.05, 80% power, and clinically relevant hazard ratios between 0.60 and 0.70, the required number of events was estimated to range from approximately 120 to 250. Given the expected discharge rate close to 100%, this corresponded approximately to the same number of participants.

## 3. Results

### 3.1. Main Features of the Study Cohort

A total of 132 pregnant women were included in the analysis and followed until hospital discharge after delivery. The median hospital stay was 4 days, and above-median hospitalization duration, operationally defined as >4 days, occurred in 44 patients (33.3%). The median age and BMI were 36.0 years and 30.8 kg/m^2^, respectively. A history of previous smoking was present in 12.1% of participants, while DM was observed in 6.8%. Chronic AHT was reported in 41.7% of patients, and 15.2% had a history of preeclampsia. Regarding obstetric characteristics, 37.1% of women were primiparous. Gestational hypertension developed in 54.5% of the cohort, and preeclampsia was diagnosed in 31.8%. Preterm delivery occurred in 26.5% of cases. The median gestational age at ABPM assessment was 12 weeks. All results are described in Table 1.

Compared with women who did not undergo ABPM, women included in the ABPM cohort showed a higher cardiovascular risk profile, with a higher age, body mass index, office systolic and diastolic blood pressure, and higher prevalence of chronic hypertension, previous preeclampsia, chronic kidney disease, and antihypertensive treatment (Supplementary Table S1). These findings support the clinically selected nature of the study population.

Median office SBP, DBP and MAP were 134.5 mmHg, 81.0 mmHg and 98.0 mmHg, respectively. On 24 h ambulatory monitoring, median 24-hSBP and 24-hDBP were 122.0 mmHg and 73.0 mmHg, respectively, whereas median 24-hMAP and 24-hPP values were 88.7 mmHg and 49.0 mmHg, respectively. All results are described in Table 2 and extended in Supplementary Table S2.

### 3.2. Univariate Analysis According to Length of Hospital Stay

Baseline clinical characteristics were broadly comparable between groups. No significant differences were observed in age, body mass index, smoking status, diabetes, parity, or previous preeclampsia. However, several pregnancy-related conditions were significantly associated with prolonged hospitalization. Gestational hypertension was more frequent among patients with a longer hospital stay (75.0% vs. 44.3%, *p* = 0.001), as was preeclampsia (61.4% vs. 17.0%, *p* < 0.001). Preterm delivery was markedly more common in this group (50.0% vs. 14.8%, *p* < 0.001), along with a higher rate of delivery interventions (*p* = 0.016). A non-significant trend was observed for previous hypertension (*p* = 0.094). Circadian blood pressure patterns were not associated with hospitalization duration (Table 1).

**Figure 1:**
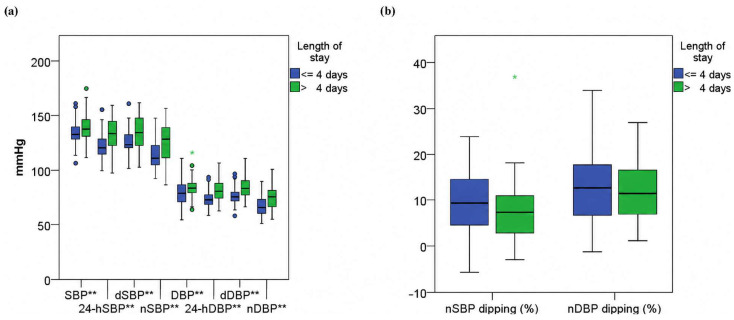
**Comparison of selected clinical and blood pressure-derived variables according to hospitalization duration.** Women were classified as having ≤4 days or >4 days of hospitalization after delivery, according to the operational cohort-specific definition of above-median hospitalization duration. Blue and green boxes correspond to the ≤4 days and >4 days groups, respectively. In the boxplots, circles indicate outliers located more than 1.5 times the interquartile range from the box, whereas asterisks indicate extreme values located more than 3 times the interquartile range from the box. (**a**) Office and out-of-office blood pressure indices; and (**b**) nighttime blood pressure dipping. Results marked with ** and * reached a *p*-value of less than 0.05 and 0.1. The numerical values of all indices are shown in Table 2. BP—blood pressure; SBP—office systolic BP; 24-hSBP—24 h SBP; dSBP—daytime SBP; nSBP—nighttime SBP; DBP—office diastolic BP; 24-hDBP—24 h DBP; dDBP—daytime DBP; nDBP—nighttime DBP; mmHg—millimeter of mercury; %—percentage.

Regarding quantitative variables, office BP values were modestly higher in patients with prolonged hospitalization, including SBP (*p* = 0.035), DBP (*p* = 0.012), and MAP (*p* = 0.010). In contrast, 24 h ABPM indices showed stronger and more consistent associations. Patients with prolonged hospitalization exhibited significantly higher 24-hSBP, 24-hDBP, and 24-hMAP (all *p* < 0.001), as well as higher daytime and nighttime BP values. The association with 24-hPP was more modest (*p* = 0.011). No significant differences were observed in heart rate or laboratory parameters (Table 2). Figure 1 presents a comparison of the main individual indices derived from 24 h ABPM.

### 3.3. Binary Logistic Regression Analysis for Prolonged Hospitalization

Binary logistic regression models were constructed to identify independent predictors of prolonged hospitalization. Potential confounders included age, BMI, DM, smoking status, gestational hypertension, circadian BP profile, preeclampsia, type of delivery, preterm delivery, uric acid levels, and angiogenic markers.

In models including office BP parameters, neither MAP nor PP remained in the final model after variable selection, indicating limited predictive value of conventional BP measurements. In contrast, ABPM-based models demonstrated independent associations with prolonged hospitalization. Higher quartiles of 24-hMAP were associated with increased odds of prolonged hospital stay (OR 1.64, 95% CI 1.05–2.55, *p* = 0.029), while higher quartiles of 24-hPP also showed a significant association (OR 1.71, 95% CI 1.06–2.74, *p* = 0.027). These associations remained significant after adjustment for key clinical variables, including preterm delivery, preeclampsia, and gestational hypertension. All results are described in Table 3.

Bootstrap resampling supported the stability of the associations for both 24-hMAP (B = 0.493, 95% CI 0.056–1.058, OR 1.64, 95% CI 1.06–2.88) and 24-hPP (B = 0.534, 95% CI 0.024–1.199, OR 1.71, 95% CI 1.02–3.32). Full bootstrap results are provided in Supplementary Table S3.

### 3.4. Discriminative Performance of 24 h MAP and 24-hPP for Prolonged Hospitalization

ROC curve analysis demonstrated good discriminative performance across all models. The clinical model without office BP indices, which were not retained after variable selection, showed an AUC of 0.836 (95% CI 0.765–0.907). Inclusion of 24-hMAP increased the AUC to 0.866 (95% CI 0.801–0.931), whereas incorporation of 24-hPP yielded an AUC of 0.859 (95% CI 0.791–0.926) (all *p* < 0.001).

**Figure 2:**
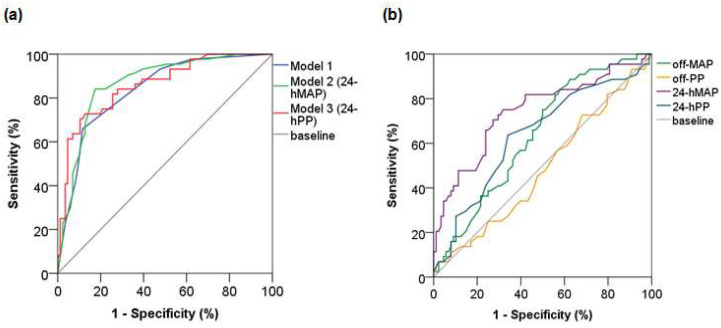
Receiver operating characteristic (ROC) curves for above-median hospitalization (>4 days). ROC curves are presented as exploratory analyses of apparent discriminative performance within the study cohort. (**a**) ROC curves for three multivariable logistic regression models: a clinical model without blood pressure indices (Model 1, blue line), a model including 24 h mean arterial pressure (24-hMAP; Model 2, green line), and a model including 24 h pulse pressure (24-hPP; Model 3, red line). The area under the curve (AUC) was 0.836 (SE 0.036; 95% CI 0.765–0.907) for Model 1, 0.866 (SE 0.033; 95% CI 0.801–0.931) for Model 2, and 0.859 (SE 0.035; 95% CI 0.791–0.926) for Model 3. At a predefined false-positive rate of 30%, the 24-hMAP model achieved a sensitivity of approximately 90%. In contrast, at a predefined false-negative rate of 30%, the 24-hPP model achieved a specificity approaching 90%. (**b**) ROC curves for individual office and ambulatory blood pressure-derived indices. The AUC values were 0.637 (SE 0.049; 95% CI 0.542–0.733; *p* = 0.010) for office MAP, 0.484 (SE 0.054; 95% CI 0.379–0.588; *p* = 0.759) for office PP, 0.743 (SE 0.048; 95% CI 0.648–0.837; *p* < 0.001) for 24-hMAP, and 0.636 (SE 0.052; 95% CI 0.533–0.738; *p* = 0.011) for 24-hPP. ROC—receiver operating characteristic; AUC—area under the curve; SE—standard error; CI—confidence interval; MAP—mean arterial pressure; PP—pulse pressure; 24-hMAP—24 h mean arterial pressure; 24-hPP—24 h pulse pressure.

The model incorporating 24-hMAP showed the highest overall discrimination. At a threshold corresponding to approximately a 30% false-positive rate, this model achieved sensitivity close to 90%, indicating a strong ability to identify patients at risk of prolonged hospitalization. In contrast, the model including 24-hPP demonstrated a complementary profile: at a threshold corresponding to approximately 30% false-negative rate (sensitivity ~70%), specificity approached 90%, suggesting improved ability to rule out prolonged hospitalization. Exploratory ROC analyses of individual BP-derived indices showed higher discrimination for ABPM-derived parameters compared with office BP measurements. At a predefined false-positive rate of 30%, sensitivities were 41% for office MAP, 73% for 24-hMAP (cutoff 91.5 mmHg), 25% for office PP, and 50% for 24-hPP (cutoff 51.0 mmHg). At a predefined false-negative rate of 30%, specificities were 88% for office MAP, 95% for 24-hMAP (cutoff 105 mmHg), 64% for office PP, and 83% for 24-hPP (cutoff 55 mmHg). These exploratory thresholds should be interpreted cautiously because they were not adjusted for relevant obstetric covariates and were not intended as clinically validated decision thresholds. These results are extended in Figure 2.

### 3.5. Kaplan–Meier Analysis of Time to Hospital Discharge

**Figure 3:**
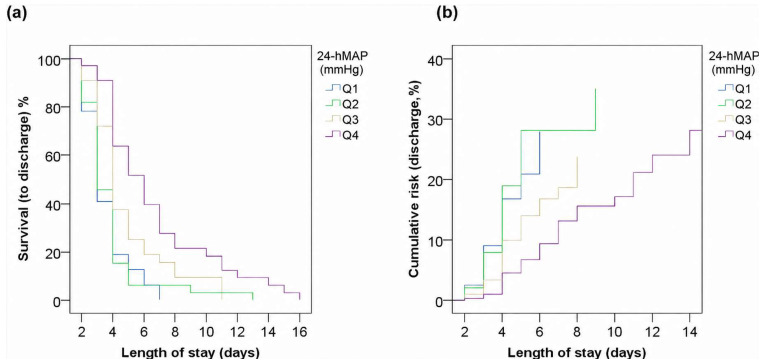
Kaplan–Meier analysis of time to hospital discharge according to 24 h mean arterial pressure. Kaplan–Meier curves showing (**a**) survival probability (i.e., probability of remaining hospitalized) and (**b**) cumulative risk of hospital discharge according to quartiles of 24 h mean arterial pressure (MAP) measured during pregnancy. Patients in higher MAP quartiles exhibited a lower probability of discharge over time and a slower increase in cumulative discharge rates, indicating longer hospital stays. A progressive separation of curves across quartiles suggests a dose–response relationship between increasing MAP and delayed discharge. Differences between groups were assessed using the log-rank test. Median length of stay increased from 3.0 days in the lowest quartile (SE 0.23; 95% CI 2.55–3.45) to 6.0 days in the highest quartile (SE 0.70; 95% CI 4.63–7.38), suggesting a dose–response relationship. Differences between groups were statistically significant (log-rank *p* < 0.05).

Kaplan–Meier curves were constructed to evaluate time to hospital discharge according to quartiles of 24-hMAP and 24-hPP. For 24-hMAP, a clear separation between curves was observed, with progressively lower discharge probabilities across increasing quartiles. Median length of stay increased from 3.0 days in the lowest quartile (SE 0.23; 95% CI 2.55–3.45) to 6.0 days in the highest quartile (SE 0.70; 95% CI 4.63–7.38), suggesting a dose–response relationship. Differences between groups were statistically significant (log-rank *p* < 0.05). The results are provided in Figure 3.

For 24-hPP, a less consistent pattern was observed. Median length of stay ranged from 3.0 to 4.0 days across quartiles (Q1: 4.0 days [SE 0.16; 95% CI 3.69–4.31], Q2: 3.0 days [SE 0.27; 95% CI 2.48–3.53], Q3: 4.0 days [SE 0.45; 95% CI 3.12–4.88], and Q4: 4.0 days [SE 0.60; 95% CI 2.83–5.17]). Although some separation between curves was observed, differences were less pronounced compared to MAP.

### 3.6. Time-to-Event Analysis Using Cox Proportional Hazards Models

A multivariable Cox proportional hazards model was used to evaluate factors associated with time to hospital discharge. The event of interest was hospital discharge, and time was defined as length of hospital stay in days. Consistent with the Kaplan–Meier analysis, higher quartiles of 24-hMAP were associated with a lower probability of discharge over time. After adjustment for potential confounders, 24-hMAP remained independently associated with time to discharge (HR 0.75, 95% CI 0.62–0.89, *p* = 0.001). Among clinical variables, preeclampsia (HR 0.37, 95% CI 0.24–0.60, *p* < 0.001) and preterm delivery (HR 0.42, 95% CI 0.25–0.72, *p* = 0.002) were also independently associated with delayed discharge. Age showed a modest inverse association (HR 0.82, *p* = 0.019), while other covariates were not statistically significant. All results are shown in Table 4.

## 4. Discussion

In this prospective study, several composite 24 h ABPM indices were independently associated with hospital-related outcomes. Both 24-hMAP and 24-hPP were associated with prolonged hospitalization, whereas only 24-hMAP remained independently associated with time to hospital discharge. In contrast, office BP measurements were not independently associated with these outcomes.

The present results should be interpreted within the clinical referral pathway of the study. ABPM was performed as part of a targeted cardiovascular risk assessment in pregnant women with baseline features suggesting increased cardiovascular risk, rather than as population-based screening. This explains the high prevalence of chronic hypertension, gestational hypertension, obesity, and preeclampsia observed in the cohort. Accordingly, the results apply primarily to clinically selected higher-risk pregnant women and should not be directly extrapolated to routine, unselected, or low-risk obstetric populations.

These findings support that composite ABPM indices may better reflect the hemodynamic burden associated with adverse clinical trajectories in pregnancy than conventional office BP measurements [27,28]. Mean arterial pressure, as a composite and linear measure of overall SBP and DBP levels, captures both systolic and diastolic components and therefore provides a more stable estimate of tissue perfusion pressure [29]. This may explain its stronger and more consistent association with hospitalization outcomes. Additionally, MAP is less affected by short-term variability and measurement conditions compared to isolated systolic or diastolic values, further reinforcing its utility as a global hemodynamic marker [30]. From a pathophysiological perspective, sustained elevations in MAP may reflect increased vascular resistance and impaired cardiovascular adaptation to pregnancy, both of which have been linked to adverse maternal outcomes [31].

Our findings support that the impact of MAP extends beyond a static association with prolonged hospitalization. Higher MAP values were associated with a reduced probability of discharge at any given time point, indicating a sustained effect over the entire hospitalization period. This reinforces the concept that hemodynamic burden influences not only the occurrence but also the temporal dynamics of recovery.

Pulse pressure, in contrast, reflects arterial stiffness and vascular compliance. Increased PP may indicate early vascular aging and reduced arterial elasticity, which are associated with endothelial dysfunction and impaired placental perfusion [32]. These alterations may contribute to a more complex clinical profile and a higher likelihood of prolonged hospitalization. However, the weaker association observed in time-to-event analyses suggests that PP may capture a different dimension of CVR, potentially related more to baseline vascular condition than to dynamic changes influencing recovery and discharge [33,34].

In contrast to the 24-hMAP findings, the association observed for 24-hPP should be interpreted with greater caution. The 24-hPP logistic model retained six predictors for 44 outcome events, resulting in a lower events-per-variable ratio (EPV ≈ 7.3). Therefore, the 24-hPP findings should be regarded as exploratory and less robust than the more consistent associations observed for 24-hMAP.

The relationship between these composite ABPM indices and time to hospital discharge remains scarcely explored in the literature. One possible explanation for the observed results is that patients with a higher BP burden may require prolonged monitoring and therapeutic adjustments before meeting discharge criteria. Additionally, these patients may represent a subgroup with more severe or complex clinical presentations, including higher rates of preeclampsia and preterm delivery, which inherently contribute to longer hospital stays [35,36,37].

From a clinical perspective, hospital-related outcomes such as length of stay provide valuable complementary information to traditional obstetric endpoints. While previous studies have focused on the role of ABPM indices in predicting hypertensive complications, our results suggest that these parameters may also contribute to a broader diagnostic–prognostic framework, including healthcare utilization and recovery patterns [38,39,40]. These findings should be interpreted as exploratory and hypothesis-generating. Further studies in larger and externally validated cohorts are required before ABPM-derived hemodynamic indices can be considered for clinical decision-making regarding hospitalization-related outcomes at delivery.

### Limitations and Strengths

This study has several limitations that should be considered when interpreting the findings: (1) Although prospectively designed, it was conducted at a single center and included a relatively modest sample size, which may have limited statistical power, particularly for smaller effect sizes and multivariable modeling. (2) Residual confounding cannot be excluded. Antihypertensive treatment may influence ABPM-derived indices and also reflect higher baseline cardiovascular risk. Given the limited number of treated women and outcome events, treatment was not included in the final multivariable models, and residual confounding by indication or treatment changes cannot be excluded. (3) Hospitalization duration should be interpreted as a composite healthcare-related outcome rather than as a diagnosis-specific endpoint. The >4-day threshold represents an operational, cohort-specific definition of above-median hospitalization duration and should not be considered a universal clinical threshold. (4) Some covariates included in the multivariable models, such as gestational hypertension, preeclampsia, preterm delivery, and delivery intervention, may occur after ABPM assessment and may therefore partly act as mediators. Accordingly, the models should be interpreted as exploratory explanatory analyses rather than as estimates of the total causal effect of ABPM-derived indices. (5) The categorization of continuous ABPM-derived variables into quartiles may have reduced statistical power and resulted in some loss of information, although this strategy improved interpretability and avoided assuming linearity. (6) The ROC analyses should be interpreted with caution because performance estimates were derived from the same cohort used to build the models. Bootstrap resampling assessed internal stability but does not replace external validation.

Despite these limitations, the study has notable strengths, including its prospective design, standardized early-pregnancy ABPM assessment, and the integration of regression, ROC, bootstrap, and time-to-event approaches in a clinically characterized higher-risk pregnancy cohort.

## 5. Conclusions

Composite ABPM indices provide clinically relevant information beyond conventional office measurements in hypertensive pregnancies. These findings support the concept that integrated hemodynamic markers, particularly those reflecting overall pressure load and vascular properties, may contribute to a more comprehensive assessment of maternal cardiovascular status. Incorporating ABPM-derived parameters into early evaluation strategies could improve risk stratification and inform clinical decision-making, not only for traditional obstetric outcomes but also for healthcare-related endpoints such as hospitalization patterns. Further studies are warranted to validate these observations and to explore their potential role in guiding individualized management approaches.

## Figures and Tables

**Table 1:** General findings and comparisons between the groups related to prolonged hospitalization. Qualitative variables.

Variable	Total *n* = 132	≤4 Days ^1^ *n* = 88	>4 Days *n* = 44	*p*-Value
Primiparity	49 (37.1)	30 (34.1)	19 (43.2)	0.343
Nº of births (>1)	15 (11.4)	12 (13.6)	3 (6.8)	0.383
Miscarriages (>1)	5 (3.8)	2 (2.3)	3 (6.8)	0.333
Previous smoking ^2^	16 (12.1)	9 (10.2)	7 (15.9)	0.400
Diabetes mellitus	9 (6.8)	6 (6.8)	3 (6.8)	1.000
Chronic hypertension ^3^	55 (41.7)	32 (36.4)	23 (52.3)	0.094
Previous preeclampsia	20 (15.2)	11 (12.5)	9 (20.5)	0.303
Gestational hypertension	72 (54.5)	39 (44.3)	33 (75.0)	0.001
Non-dipper	78 (59.1)	53 (60.2)	25 (56.8)	0.712
Preeclampsia	42 (31.8)	15 (17.0)	27 (61.4)	<0.001
Delivery (intervention) ^4^	70 (53.0)	40 (45.5)	30 (68.2)	0.016
Preterm delivery	35 (26.5)	13 (14.8)	22 (50.0)	<0.001
Antihypertensive drugs	22 (16.7)	8 (9.2)	14 (31.8)	<0.001

^1^ Patient groups according to the presence of prolonged hospitalization (>4 days); ^2^ former smokers for more than 6 months at the time of recruitment. The results are expressed as number and percentage. ^3^ Chronic hypertension refers to pre-existing hypertension, defined as hypertension diagnosed before pregnancy or before 20 weeks of gestation, or antihypertensive treatment for previously diagnosed hypertension before pregnancy. ^4^ Delivery intervention refers to cesarean procedure during admission for delivery.

**Table 2:** General findings and comparisons between the groups related to prolonged hospitalization. Quantitative variables.

Variable	Total *n* = 132	≤4 Days ^1^ *n* = 88	>4 Days *n* = 44	*p*-Value
Age (years)	36.0 (8.0)	36.0 (8.8)	37.0 (8.0)	0.744
BMI (kg/m^2^)	30.8 (11.32)	31.72 (11.02)	29.07 (13.55)	0.772
SBP (mmHg)	134.5 (12.8)	132.0 (12.5)	137.0 (15.8)	0.035
DBP (mmHg)	81.0 (13.8)	78.0 (16.0)	83.0 (9.5)	0.012
MAP (mmHg)	98.0 (13.75)	96.17 (13.17)	100.83 (11.08)	0.010
PP (mmHg)	53.5 (14.5)	54.0 (13.75)	53.0 (14.5)	0.759
24-hSBP (mmHg)	122.0 (18.0)	120.0 (14.0)	132.5 (23.3)	<0.001
24-hDBP (mmHg)	73.0 (11.8)	72.0 (9.0)	80.0 (15.0)	<0.001
24-hMAP (mmHg)	88.67 (12.33)	87.33 (9.50)	96.00 (17.00)	<0.001
24-hPP (mmHg)	49.0 (10.0)	47.0 (8.75)	51.0 (11.0)	0.011
Hemoglobin (mg/dL)	12.0 (1.3)	12.0 (1.4)	12.0 (1.0)	0.614
Platelets (×10^9^/L)	239.0 (87.0)	234.0 (90.0)	259.5 (92.25)	0.095
Leukocytes (×10^9^/L)	9.16 (3.3)	9.02 (3.66)	9.32 (2.82)	0.263
FPG (mg/dL)	78.0 (13.3)	78.0 (14.0)	78.0 (15.0)	0.892
Uric acid (mg/dL)	4.1 (1.5)	4.10 (1.40)	4.45 (1.55)	0.079
sFlt-1/PlGF ratio	7.0 (22.0)	6.5 (20.0)	9.0 (45.0)	0.076

^1^ Patient groups according to the presence of prolonged hospitalization (>4 days). Results are expressed as median and interquartile range. BMI—body mass index; BP—blood pressure; SBP—systolic blood pressure; DBP—diastolic blood pressure; MAP—mean arterial pressure; PP—pulse pressure; 24-hSBP—24 h systolic blood pressure; 24-hDBP—24 h diastolic blood pressure; 24-hMAP—24 h mean arterial pressure; 24-hPP—24 h pulse pressure; FPG—fasting plasma glucose; sFlt-1—soluble fms-like tyrosine kinase-1; PlGF—placental growth factor; kg—kilogram; m—meter; mmHg—millimeter of mercury; mg—milligram; dL—deciliter; L—liter.

**Table 3:** Binary logistic regression models for prolonged hospitalization (>4 days).

Variable	B	SE	*p*-Value	Exp(B) ^1^	95% CI (Lower–Upper)
***Model 1 (including office MAP and office PP)***					
Preterm delivery	1.901	0.617	0.002	6.690	1.997–22.418
Preeclampsia	1.758	0.507	0.001	5.802	2.148–15.672
Gestational hypertension	1.773	0.560	0.002	5.887	1.964–17.645
Constant	−2.903				
***Model 2 (including 24-hMAP)***					
Preterm delivery	1.491	0.573	0.009	4.439	1.444–13.648
Preeclampsia	1.610	0.502	0.001	5.003	1.872–13.374
Gestational hypertension	1.455	0.557	0.009	4.286	1.437–12.778
24-hMAP (quartiles)	0.493	0.225	0.029	1.637	1.052–2.546
Constant	−3.422				
***Model 3 (including 24-hPP)***					
Age (quartiles)	0.389	0.231	0.092	1.476	0.938–2.321
Preterm delivery	2.023	0.650	0.002	7.561	2.113–27.049
Preeclampsia	2.216	0.587	<0.001	9.167	2.902–28.955
Gestational hypertension	1.435	0.570	0.012	4.198	1.374–12.830
sFlt-1/PlGF (quartiles)	−0.450	0.263	0.086	0.637	0.381–1.067
24-hPP (quartiles)	0.534	0.242	0.027	1.705	1.062–2.738
Constant	−3.638				

^1^ Binary logistic regression models for prolonged hospitalization (>4 days). Multivariable logistic regression analyses were performed to identify independent predictors of prolonged hospitalization. All models incorporated data from 132 pregnant women ensuring no missing data. The variables to be controlled were age, body mass index, diabetes, former smoking, preterm delivery, delivery (intervention), gestational hypertension, preeclampsia, non-dipper profile, uric acid and sFlt-1/PlGF ratio. Continuous variables were categorized into quartiles to account for potential non-linear associations. For the final models, a backward stepwise selection procedure (Wald method) was applied, retaining variables with *p* < 0.10. Three models were constructed: ***Model 1 (EPV: 14.3)*** included clinical variables and office blood pressure indices (mean arterial pressure and pulse pressure), which were not retained in the final model; ***Model 2 (EPV: 11)*** incorporated 24 h mean arterial pressure; and ***Model 3 (EPV: 7.3)*** incorporated 24 h pulse pressure. Results are presented as regression coefficients (B), standard error (SE), *p*-values, odds ratios (Exp(B)), and 95% confidence intervals (CIs). For all the models: Omnibus test of model coefficients (*p*-value) < 0.05; and Hosmer–Lemeshow test (*p*-value) > 0.05. 24-hMAP—24 h mean arterial pressure; 24-hPP—24 h pulse pressure.

**Table 4:** Multivariable Cox regression model for time to hospital discharge.

Variable	B (SE)	HR (95% CI)	*p*-Value
Age (quartiles)	−0.197 (0.084)	0.82 (0.70–0.97)	0.019
Smoking	−0.536 (0.302)	0.59 (0.32–1.06)	0.075
Preeclampsia	−0.984 (0.238)	0.37 (0.24–0.60)	<0.001
Gestational hypertension	−0.386 (0.202)	0.68 (0.46–1.01)	0.056
Delivery (intervention)	−0.311 (0.187)	0.73 (0.51–1.06)	0.097
Preterm delivery	−0.865 (0.273)	0.42 (0.25–0.72)	0.002
24-hMAP (quartiles)	−0.294 (0.092)	0.75 (0.62–0.89)	0.001

Multivariable Cox proportional hazards model for time to hospital discharge. A multivariable Cox proportional hazards regression model was used to evaluate factors associated with time to hospital discharge. The event of interest was hospital discharge, and time was defined as length of hospital stay in days. Variables were selected based on clinical relevance and univariable analysis, and entered into the multivariable model. Continuous variables were categorized into quartiles to account for potential non-linear relationships. Results are presented as regression coefficients (B), standard error (SE), hazard ratios (HRs), and 95% confidence intervals (CI). Hazard ratios < 1 indicate a lower probability of discharge over time (i.e., longer hospitalization). The proportional hazards assumption was assessed using time-dependent covariates and graphical methods.

## Data Availability

Data is available on reasonable request from the corresponding author. In accordance with Article 18.4 of the Spanish Constitution and the Organic Law on Data Protection and Guarantee of Digital Rights (LOPDGDD) of 6 December 2018, the privacy and integrity of the individual will be protected at all times, so anonymous data are available upon reasonable request.

## Supplementary Materials

The following supporting information can be downloaded at: https://www.mdpi.com/article/10.3390/jcm15135188/s1, Figure S1. Clinical pathway leading to ABPM referral. Pregnant women were initially evaluated during first-trimester obstetric care. Women with baseline clinical features suggesting increased cardiovascular risk were referred from Obstetrics to the Hypertension and Cardiovascular Risk Unit of Internal Medicine, where office blood pressure assessment and 24-h ABPM were performed. The resulting ABPM cohort represents a clinically selected higher-risk subgroup. Women from the same institutional setting who did not undergo ABPM were used only to contextualize the referral profile and external validity and were not used as an outcome control group. Table S1: Comparison between pregnant women who underwent ambulatory blood pressure monitoring and women who did not undergo ambulatory blood pressure monitoring in the same institutional setting; Table S2: General findings and comparisons between hospitalization-duration groups. Quantitative variables (extended version); Table S3. Bootstrap validation of binary logistic regression models.

## References

[1] Wu P., Green M., Myers J.E. (2023). Hypertensive disorders of pregnancy. BMJ.

[2] Agrawal A., Wenger N.K. (2020). Hypertension During Pregnancy. Curr. Hypertens. Rep..

[3] Sanghavi M., Rutherford J.D. (2014). Cardiovascular physiology of pregnancy. Circulation.

[4] Penny J.A., Halligan A.W., Shennan A.H., Lambert P.C., Jones D.R., de Swiet M., Taylor D.J. (1998). Automated, ambulatory, or conventional blood pressure measurement in pregnancy: Which is the better predictor of severe hypertension?. Am. J. Obstet. Gynecol..

[5] Webster J. (2019). Ambulatory blood pressure monitoring in pregnancy: A better guide to risk assessment?. J. Hypertens..

[6] Fernandez-Castro I., Vazquez-Agra N., Alban-Salgado A., Sanchez-Andrade M., Lopez-Casal S., Cruces-Sande A., Seoane-Casqueiro O., Pose-Reino A., Hermida-Ameijeiras A. (2025). Evaluating an Early Risk Model for Uncomplicated Hypertension in Pregnancy Based on Nighttime Blood Pressure, Uric Acid, and Angiogenesis-Related Factors. Int. J. Mol. Sci..

[7] Rhodes C.A., Beevers D.G., Churchill D. (2018). A randomized trial of ambulatory blood pressure monitoring versus clinical blood pressure measurement in the management of hypertension in pregnancy. A feasibility study. Pregnancy Hypertens..

[8] Ayala D.E., Hermida R.C. (2013). Ambulatory blood pressure monitoring for the early identification of hypertension in pregnancy. Chronobiol. Int..

[9] Hermida R.C., Ayala D.E. (2002). Prognostic value of office and ambulatory blood pressure measurements in pregnancy. Hypertension.

[10] Wen T., Yu V.X., Wright J.D., Goffman D., Attenello F., Mack W.J., D’alton M., Friedman A.M. (2020). Postpartum length of stay and risk for readmission among women with preeclampsia. J. Matern Fetal Neonatal Med..

[11] Mancia G., Kreutz R., Brunström M., Burnier M., Grassi G., Januszewicz A. (2023). 2023 ESH Guidelines for the management of arterial hypertension The Task Force for the management of arterial hypertension of the European Society of Hypertension: Endorsed by the International Society of Hypertension (ISH) and the European Renal Association (ERA). J. Hypertens..

[12] Visseren F.L.J., Mach F., Smulders Y.M., Carballo D., Koskinas K.C., Bäck M., Benetos A., Biffi A., Boavida J.M., Capodanno D. (2021). 2021 ESC Guidelines on cardiovascular disease prevention in clinical practice. Eur. Heart J..

[13] El Consumo de Alcohol y su Salud|Hojas Informativas|Alcohol|CDC 2022. https://www.cdc.gov/alcohol/hojas-informativas/consumo-alcohol-salud.html.

[14] Flegal K.M. (2017). Body-mass index and all-cause mortality. Lancet.

[15] Stergiou G.S., O’Brien E., Myers M., Palatini P., Parati G., Kollias A., Birmpas D., Kyriakoulis K., Bountzona I., Stambolliu E. (2019). STRIDE BP international initiative for accurate blood pressure measurement: Systematic review of published validation studies of blood pressure measuring devices. J. Clin. Hypertens..

[16] Magee L.A., Smith G.N., Bloch C., Côté A.-M., Jain V., Nerenberg K., von Dadelszen P., Helewa M., Rey E. (2022). Guideline No. 426: Hypertensive Disorders of Pregnancy: Diagnosis, Prediction, Prevention, and Management. J. Obstet. Gynaecol. Can..

[17] ElSayed N.A., Aleppo G., Aroda V.R., Bannuru R.R., Brown F.M., Bruemmer D., Collins B.S., Hilliard M.E., Isaacs D., Johnson E.L. (2023). 2. Classification and Diagnosis of Diabetes: Standards of Care in Diabetes-2023. Diabetes Care.

[18] Vazquez-Agra N., Cruces-Sande A., Barbosa-Gouveia S., Lopez-Paz J.-E., Camafort M., Casariego-Vales E., Pose-Reino A., Hermida-Ameijeiras A. (2024). Assessing the relationship between lipoprotein(a) levels and blood pressure among hypertensive patients beyond conventional measures. An observational study. Sci. Rep..

[19] Vazquez-Agra N., Barrera-Lopez L., Marques-Afonso A.-T., Cruces-Sande A., Lopez-Paz J.-E., Pose-Reino A., Hermida-Ameijeiras A. (2025). Assessing the relationship between short-term blood pressure variability and glycation profile in young and middle-aged nondiabetic hypertensive individuals. J. Hypertens..

[20] Mancia G., Verdecchia P. (2015). Clinical value of ambulatory blood pressure: Evidence and limits. Circ. Res..

[21] O’Brien E., Sheridan J., O’Malley K. (1988). Dippers and non-dippers. Lancet.

[22] Harrell F.E., Harrell F.E. (2015). Multivariable Modeling Strategies. Regression Modeling Strategies: With Applications to Linear Models, Logistic and Ordinal Regression, and Survival Analysis.

[23] Avolio A.P., Kuznetsova T., Heyndrickx G.R., Kerkhof P.L.M., Li J.K.-J. (2018). Arterial Flow, Pulse Pressure and Pulse Wave Velocity in Men and Women at Various Ages. Adv. Exp. Med. Biol..

[24] Safar M.E., Levy B.I., Struijker-Boudier H. (2003). Current perspectives on arterial stiffness and pulse pressure in hypertension and cardiovascular diseases. Circulation.

[25] Peduzzi P., Concato J., Kemper E., Holford T.R., Feinstein A.R. (1996). A simulation study of the number of events per variable in logistic regression analysis. J. Clin. Epidemiol..

[26] Schoenfeld D.A. (1983). Sample-Size Formula for the Proportional-Hazards Regression Model. Biometrics.

[27] Salazar M.R., Espeche W.G., Leiva Sisnieguez C.E., Minetto J., Balbín E., Soria A., Yoma O., Prudente M., Torres S., Grassi F. (2021). Nocturnal hypertension and risk of developing early-onset preeclampsia in high-risk pregnancies. Hypertens. Res..

[28] Fang Y., Zuo L., Duan H., Huang C., Wen J., Yang Q., Han C., Lv L., Zhou X. (2025). Hypertension phenotypes and adverse pregnancy outcome-related office and ambulatory blood pressure thresholds during pregnancy: A retrospective cohort study. Hypertens. Res..

[29] DeMers D., Wachs D. (2026). Physiology, Mean Arterial Pressure.

[30] Kandil H., Soliman A., Alghamdi N.S., Jennings J.R., El-Baz A. (2023). Using Mean Arterial Pressure in Hypertension Diagnosis versus Using Either Systolic or Diastolic Blood Pressure Measurements. Biomedicines.

[31] Jin M., Liu X., Liu X., Wu Y., Zhang Y., Zhang L., Li Z., Ye R., Li N. (2024). Association of pre-/early pregnancy high blood pressure and pregnancy outcomes: A systemic review and meta-analysis. J. Matern. Fetal Neonatal Med..

[32] Lee H.-Y., Oh B.-H. (2010). Aging and arterial stiffness. Circ. J..

[33] Selvaraj S., Steg P.G., Elbez Y., Sorbets E., Feldman L.J., Eagle K.A., Ohman E.M., Blacher J., Bhatt D.L. (2016). Pulse Pressure and Risk for Cardiovascular Events in Patients With Atherothrombosis: From the REACH Registry. J. Am. Coll. Cardiol..

[34] Laskey W.K., Wu J., Schulte P.J., Hernandez A.F., Yancy C.W., Heidenreich P.A., Bhatt D.L., Fonarow G.C. (2016). Association of Arterial Pulse Pressure With Long-Term Clinical Outcomes in Patients With Heart Failure. JACC Heart Fail..

[35] Szczepaniak-Chicheł L., Lipski D., Uruski P., Tykarski A. (2025). Differences between office, home, and 24-hour ambulatory blood pressure monitoring in pregnant women with preeclampsia and uncomplicated hypertension. Pol. Arch. Intern Med..

[36] Li X., Lan N., Guo Y., Pei M., Jiang Y., Zou Y. (2026). Beyond clinic readings: Twenty-four hour ambulatory blood pressure monitoring profiling enhances preterm delivery risk stratification in hypertensive pregnancies. Int. J. Gynaecol. Obstet..

[37] Pant A., Mukherjee S., Watts M., Marschner S., Mihailidou A.S., O’Brien J., Beale A., Chow C.K., Zaman S. (2025). Detection of hypertension and blood pressure phenotypes using ambulatory blood pressure monitoring in women with past hypertensive disorders of pregnancies. Pregnancy Hypertens..

[38] Rajkumar T., Hennessy A., Makris A. (2025). Remote blood pressure monitoring in women at risk of or with hypertensive disorders of pregnancy: A systematic review and meta-analysis. Int. J. Gynaecol. Obstet..

[39] Chappell L.C., Tucker K.L., Galal U., Yu L.-M., Campbell H., Rivero-Arias O., Allen J., Band R., Chisholm A., Crawford C. (2022). Effect of Self-monitoring of Blood Pressure on Blood Pressure Control in Pregnant Individuals With Chronic or Gestational Hypertension: The BUMP 2 Randomized Clinical Trial. JAMA.

[40] Moustafa A.S.Z., Yimer W., Perry A., Solis L., Belk S., Morris R., Spencer S.-K., Rana S., Wallace K. (2024). Report from a text-based blood pressure monitoring prospective cohort trial among postpartum women with hypertensive disorders of pregnancy. BMC Pregnancy Childbirth.

