# Supplementary material for: Twenty-Four-Hour Mean Arterial Pressure and Pulse Pressure Are Associated with Hospitalization Duration at Delivery in Pregnant Women Referred for Cardiovascular Risk Assessment
Source: J Clin Med. 2026 Jul 2;15(13):5188. doi: 10.3390/jcm15135188 (PMC13362545; doi:10.3390/jcm15135188)
Supplement: Supplementary file 1 [file jcm-15-05188-s001.zip › Supplementary_Table_S2.pdf]

Supplementary Table S2. General findings and comparisons between hospitalization-duration groups. Quantitative variables (extended version).

Variable	Total (n=132)	≤4 days (n=88)	>4 days (n=44)	p-value
Age, years	36.0 (8.0)	36.0 (8.8)	37.0 (8.0)	0.744
BMI, kg/m^2	30.8 (11.32)	31.72 (11.02)	29.07 (13.55)	0.772
Office SBP, mmHg	134.5 (12.8)	132.0 (12.5)	137.0 (15.8)	0.035
Office DBP, mmHg	81.0 (13.8)	78.0 (16.0)	83.0 (9.5)	0.012
Heart rate, bpm	90.0 (18.0)	90.0 (18.0)	89.5 (17.8)	0.963
Office MAP, mmHg	98.0 (13.75)	96.17 (13.17)	100.83 (11.08)	0.010
Office PP, mmHg	53.5 (14.5)	54.0 (13.75)	53.0 (14.5)	0.759
24-h SBP, mmHg	122.0 (18.0)	120.0 (14.0)	132.5 (23.3)	<0.001
24-h DBP, mmHg	73.0 (11.8)	72.0 (9.0)	80.0 (15.0)	<0.001
24-h MAP, mmHg	88.67 (12.33)	87.33 (9.50)	96.00 (17.00)	<0.001
24-h PP, mmHg	49.0 (10.0)	47.0 (8.75)	51.0 (11.0)	0.011
Daytime SBP, mmHg	127.0 (15.0)	122.0 (13.8)	133.5 (25.0)	<0.001
Daytime DBP, mmHg	77.0 (11.8)	75.0 (8.0)	83.0 (13.8)	<0.001
Nighttime SBP, mmHg	115.0 (23.5)	110.0 (18.5)	128.0 (28.5)	<0.001
Nighttime DBP, mmHg	66.5 (16.0)	65.0 (13.5)	75.0 (15.5)	<0.001
Nighttime SBP dipping, %	8.15 (9.4)	8.78 (10.0)	7.21 (8.3)	0.148
Nighttime DBP dipping, %	12.28 (10.3)	12.50 (11.1)	11.25 (9.8)	0.390
24-h heart rate, bpm	83.0 (12.5)	83.0 (12.0)	83.0 (14.0)	0.598
Daytime heart rate, bpm	85.0 (13.0)	85.0 (12.8)	86.5 (15.0)	0.744
Nighttime heart rate, bpm	77.0 (11.0)	77.0 (11.8)	79.0 (12.5)	0.096
Hemoglobin, mg/dL	12.0 (1.3)	12.0 (1.4)	12.0 (1.0)	0.614
Platelets, x10^9/L	239.0 (87.0)	234.0 (90.0)	259.5 (92.25)	0.095
Leukocytes, x10^9/L	9.16 (3.3)	9.02 (3.66)	9.32 (2.82)	0.263
Fasting plasma glucose, mg/dL	78.0 (13.3)	78.0 (14.0)	78.0 (15.0)	0.892
Uric acid, mg/dL	4.1 (1.5)	4.10 (1.40)	4.45 (1.55)	0.079
sFlt-1/PlGF ratio	7.0 (22.0)	6.5 (20.0)	9.0 (45.0)	0.076

Values are expressed as median (interquartile range). Patient groups are defined according to hospitalization duration after delivery; >4 days corresponds to the operational cohort-specific definition of above-median hospitalization duration. BMI, body mass index; DBP, diastolic blood pressure; MAP, mean arterial pressure; PP, pulse pressure; SBP, systolic blood pressure; sFlt-1, soluble fms-like tyrosine kinase-1; PlGF, placental growth factor.
