# Supplementary material for: Twenty-Four-Hour Mean Arterial Pressure and Pulse Pressure Are Associated with Hospitalization Duration at Delivery in Pregnant Women Referred for Cardiovascular Risk Assessment
Source: J Clin Med. 2026 Jul 2;15(13):5188. doi: 10.3390/jcm15135188 (PMC13362545; doi:10.3390/jcm15135188)
Supplement: Supplementary file 1 [file jcm-15-05188-s001.zip › Supplementary_Table_S3.pdf]

Supplementary Table S3. Bootstrap validation of binary logistic regression models.

Variable	B	Bias	SE	p-value	95% CI for B	OR	95% CI for OR
**Model including 24-hMAP quartiles**							
Preterm delivery	1.491	0.185	1.268	0.006	0.371-3.231	4.44	1.45-25.31
Gestational hypertension	1.455	0.186	1.215	0.004	0.443-3.173	4.29	1.56-23.88
Preeclampsia	1.610	0.081	0.610	0.003	0.605-2.964	5.00	1.83-19.38
24-h MAP (quartiles)	0.493	0.042	0.260	0.025	0.056-1.058	1.64	1.06-2.88
**Model including 24-hPP quartiles**							
Age (quartiles)	0.389	0.035	0.271	0.103	-0.092-0.989	1.48	0.91-2.69
Preterm delivery	2.023	0.208	1.131	0.001	0.859-3.952	7.56	2.36-52.04
sFlt-1/PlGF ratio (quartiles)	-0.450	-0.041	0.349	0.129	-1.291-0.080	0.64	0.28-1.08
Gestational hypertension	1.435	0.207	1.119	0.004	0.368-3.221	4.20	1.44-25.05
Preeclampsia	2.216	0.238	0.713	0.001	1.244-3.962	9.17	3.47-52.57
24-h PP (quartiles)	0.534	0.050	0.285	0.028	0.024-1.199	1.71	1.02-3.32

Bootstrap validation of multivariable logistic regression models evaluating the association between ambulatory blood pressure-derived indices and above-median hospitalization duration at delivery. Simple bootstrap resampling with 100 iterations was performed. Reported values include bootstrap regression coefficients (B), bias, bootstrap standard errors (SE), two-sided bootstrap p-values, 95% bootstrap confidence intervals (CI) for B, and exponentiated coefficients expressed as odds ratios (OR) with corresponding 95% CI for OR. The first model includes 24-hour mean arterial pressure (24-hMAP) quartiles, whereas the second model includes 24-hour pulse pressure (24-hPP) quartiles. The 24-hPP model should be interpreted as exploratory because of the higher number of retained predictors relative to the number of outcome events.
