# Supplementary material for: Twenty-Four-Hour Mean Arterial Pressure and Pulse Pressure Are Associated with Hospitalization Duration at Delivery in Pregnant Women Referred for Cardiovascular Risk Assessment
Source: J Clin Med. 2026 Jul 2;15(13):5188. doi: 10.3390/jcm15135188 (PMC13362545; doi:10.3390/jcm15135188)
Supplement: Supplementary file 1 [file jcm-15-05188-s001.zip › Supplementary Figure S1_.pdf]

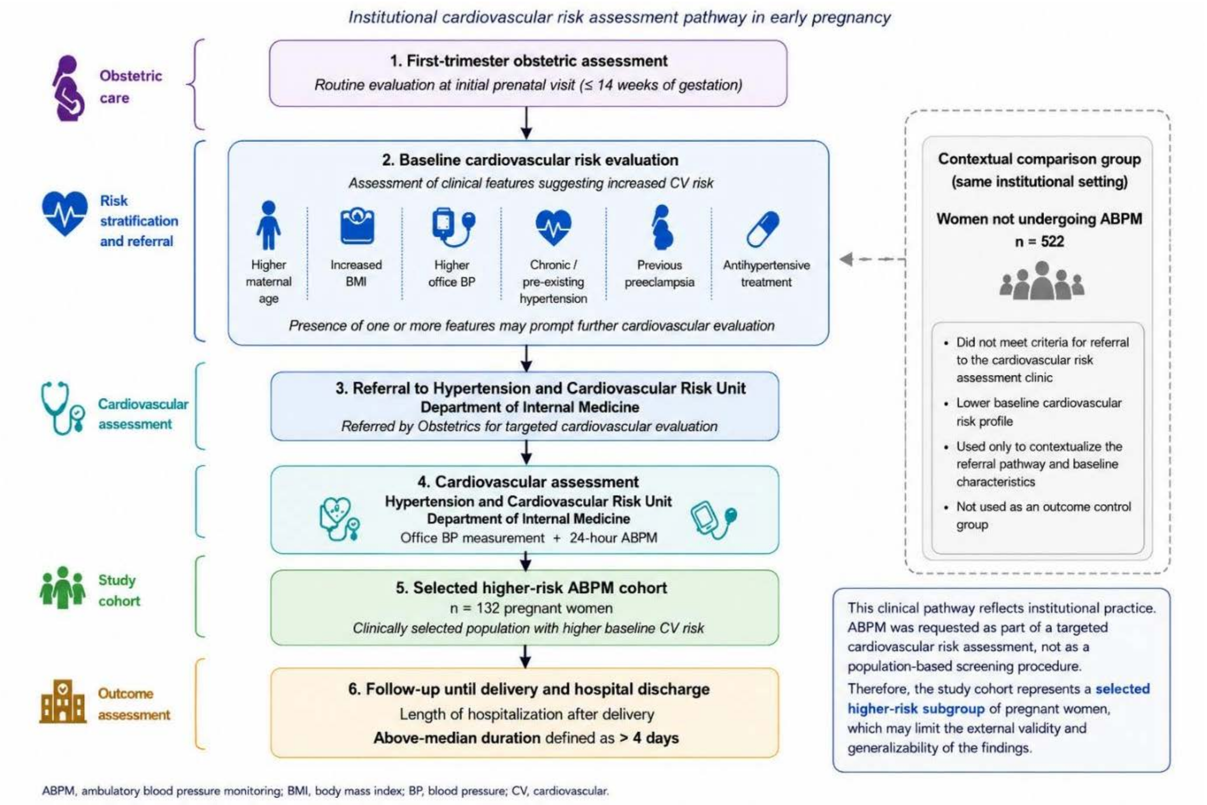

Supplementary Figure S1. Clinical pathway leading to ABPM referral. Pregnant women were initially evaluated during first-trimester obstetric care. Women with baseline clinical features suggesting increased cardiovascular risk were referred from Obstetrics to the Hypertension and Cardiovascular Risk Unit of Internal Medicine, where office blood pressure assessment and 24-hour ABPM were performed. The resulting ABPM cohort represents a clinically selected higher-risk subgroup. Women from the same institutional setting who did not undergo ABPM were used only to contextualize the referral profile and external validity and were not used as an outcome control group.
