# Supplementary material for: Twenty-Four-Hour Mean Arterial Pressure and Pulse Pressure Are Associated with Hospitalization Duration at Delivery in Pregnant Women Referred for Cardiovascular Risk Assessment
Source: J Clin Med. 2026 Jul 2;15(13):5188. doi: 10.3390/jcm15135188 (PMC13362545; doi:10.3390/jcm15135188)
Supplement: Supplementary file 1 [file jcm-15-05188-s001.zip › Supplementary_Table_S1.pdf]

Supplementary Table S1. Comparison between pregnant women who underwent ambulatory blood pressure monitoring and women who did not undergo ambulatory blood pressure monitoring in the same institutional setting.

Variable	Overall (n=654)	No ABPM (n=522)	ABPM (n=132)	p-value
**Categorical variables**				
Primiparity, yes	261/649 (40.2%)	212/517 (41.0%)	49/132 (37.1%)	0.417
Parity >1, yes	87/654 (13.3%)	72/522 (13.8%)	15/132 (11.4%)	0.463
Previous miscarriages >1, yes	47/654 (7.2%)	42/522 (8.0%)	5/132 (3.8%)	0.091
Smoking, yes	78/653 (11.9%)	62/521 (11.9%)	16/132 (12.1%)	0.944
Diabetes mellitus, yes	35/653 (5.4%)	26/521 (5.0%)	9/132 (6.8%)	0.405
Previous AHT, yes	67/654 (10.2%)	12/522 (2.3%)	55/132 (42.0%)	<0.001
Previous PE, yes	44/640 (7.0%)	24/508 (4.7%)	20/132 (15.0%)	<0.001
Antihypertensive treatment, yes	32/405 (8.0%)	10/273 (3.7%)	22/132 (16.7%)	<0.001
**Quantitative variables**				
Age, years	35.0 (8.0)	34.5 (7.0)	36.0 (8.0)	0.007
BMI, kg/m²	28.3 (9.24)	27.85 (8.93)	30.80 (11.40)	<0.001
Office SBP, mmHg	128.0 (16.0)	126.0 (15.8)	134.5 (12.8)	<0.001
Office DBP, mmHg	77.0 (16.0)	76.0 (16.0)	81.0 (13.8)	<0.001
Heart rate, bpm	89.0 (18.0)	88.0 (18.0)	90.0 (18.0)	<0.001

This comparison was performed to contextualize the selected cardiovascular risk profile of the ABPM cohort and was not used as a control group for the outcome analyses. Values are n/N (%) for categorical variables and median (IQR) for overall quantitative variables. Overall values were calculated using available denominators. p values were obtained using chi-square or Fisher exact tests for categorical variables, as appropriate, and Mann-Whitney U tests for quantitative variables. ABPM, ambulatory blood pressure monitoring; AHT, arterial hypertension; AI, variable as recorded in the source database; BMI, body mass index; DBP, diastolic blood pressure; MAP, mean arterial pressure; PE, preeclampsia; SBP, systolic blood pressure.
